# Effects of Digoxin in Heart Failure (HF) With Reduced Ejection Fraction (EF)

**DOI:** 10.7759/cureus.22778

**Published:** 2022-03-02

**Authors:** Riya R Parikh, Khushbu R Patel, Joseph V Pergolizzi, Frank Breve, Peter Magnusson

**Affiliations:** 1 Department of Pharmacy, Temple University, Philadelphia, USA; 2 Cardiology, Native Cardio Inc., Naples, USA; 3 Cardiology, School of Medical Sciences, Örebro University, Örebro, SWE; 4 Medicine, Cardiology Research Unit, Karolinska Institutet, Stockholm, SWE

**Keywords:** cv, cardiovascular, readmission, rehospitalization, mortality, reduced ejection fraction, digitalis, heart failure, digoxin, hfref

## Abstract

In this review, we evaluated the literature on the benefits and deleterious effects of digoxin in heart failure (HF) with reduced ejection fraction (EF). Although digoxin was considered an effective treatment for HF, the supporting evidence is conflicting. Before the conventional use of modern HF therapies, digoxin was widely used for symptomatic relief on these patients. Further randomized trials are required to reach a definite conclusion about its efficacy and safety in patients experiencing HF with a reduced EF (HFrEF).

## Introduction and background

Digoxin is a drug derived from the foxglove plant (*Digitalis*) and is used to treat heart failure (HF) and rate control of atrial fibrillation (AF). Before the conventional use of modern therapies for HF, some randomized clinical trials showed improvement in symptoms and lower rates of HF hospitalization through digoxin. HF is one of the most common causes of hospital admission and readmission worldwide [1]. It is a severe condition that is caused due to a low cardiac output that is insufficient in meeting the metabolic demands of the body, often resulting from structural and functional changes in the heart and leading to systolic or diastolic contractile dysfunctions. Common symptoms of HF are dyspnea, sleep disturbances, exercise intolerance, edema, and fatigue. Hypertensive heart disease and coronary artery disease are the more common risk factors [2]. Even though many HF patients are medically managed on evidence-based but complex drug therapy, there is still a high rate of morbidity, mortality, and hospital admissions and readmissions seen for HF patients. It is one of the main causes of hospitalization and high mortality rates [3,4].

Around 37.7 million people worldwide are reported to have HF [5]. This number is projected to increase due to aging and other comorbidities like type 2 diabetes mellitus, chronic kidney disease, and myocardial infarction. In the United States, approximately five million patients have been diagnosed with HF. HF accounts for a high mortality rate, with more than 80,000 deaths in the United States in 2017 [6]. HF is a costly condition [7], and public health efforts to prevent it are laudable, but efforts to treat it in a better way represent an urgent unmet medical need [6]. HF can be classified into three categories based on the left ventricular ejection fraction (EF): HF with preserved EF (HFpEF ≥ 50%), HF with reduced EF (HFrEF ≤ 40%), and HF with mid-range EF (HFmEF ≥ 41% but ≤ 49%) [5]. Acute decompensated systolic HF is also referred to as HFrEF [8]. HFrEF is conceptualized as a reactive cycle where an initial injury or malfunction results in a decreased cardiac output and triggers further malfunctions. Any myocardial injury, hypertension, abnormal loading, valvular disorders, and tachyarrhythmias may predispose a patient to HFrEF [9]. The 2021 update to HF treatment guidelines confirmed that although digoxin is indicated for treatment in HFrEF, its use in contemporary HFrEF treatment plans is confined to rate control in hypotensive patients with AF [10]. In a randomized clinical trial, patients on digoxin therapy had a lower EF and a more advanced stage of HF as seen from compromised baseline function and poorer general health [11]. Since digoxin alters cardiac output and decreases pulmonary capillary wedge pressure without increasing the heart rate or reducing blood pressure, it has demonstrated a reduction in all-cause and cardiovascular (CV) hospitalizations as seen in the Digitalis Investigation Group (DIG) trial, but no effects on all-cause and CV mortality rates [11].

However, there seems to be a dearth of retrospective analyses, and the few that are available have conflicting results and limitations relating to high low ventricular EF (LVEF) criteria for assessing HFrEF, the inclusion of tachycardia-induced cardiomyopathy due to AF, several participants on suboptimal HFrEF treatment plans, high-dose digoxin use, and use of subgroup analyses instead of the whole sample group in randomized clinical trials [12].

The 2017 American College of Cardiology/American Heart Association/Heart Failure Society of America (ACC/AHA/HFSA) focused update of the 2013 American College of Cardiology Foundation (ACCF)/AHA guideline [13] for management of HF concurred that the addition of digoxin may be considered during the initial treatment period in patients presenting severe symptoms with no symptomatic response to guideline-directed medical therapy.

Clinicians may also wait to initiate digoxin until patients show a definite response to other agents and consider digoxin for patients resistant to neurohormonal antagonist therapy. Digoxin is usually initiated and maintained at a 0.125-0.25 mg daily dose, is generally well-tolerated, and requires no loading dose. Plasma levels of 0.5-0.9 ng/mL are suggested to be efficacious, but toxicity may be observed at serum levels > 2 ng/mL [14]. The 2021 European Society of Cardiology (ESC) guidelines for diagnosis and treatment of acute and chronic HF recommend maintaining serum digoxin levels under 1.2 ng/mL due to its therapeutic window [15]. Although digoxin has been used historically and may be beneficial in certain patient groups, its narrow therapeutic index requires it to be used with clinical caution to avoid events of digoxin toxicity.

Additionally, the 2021 ESC guidelines for diagnosis and treatment of acute and chronic HF suggest a potential benefit of using digoxin to obtain ventricular rate control in patients with HFrEF and AF who cannot utilize other therapies [15]. In this review, we evaluated current literature from randomized studies to estimate the efficacy of digoxin in HFrEF concerning hospitalization events and mortality.

Methods

Between May 2020 to August 2021, a literature search was conducted. PubMed/MEDLINE, ProQuest, Google Scholar, Elsevier, and Web of Science were used primarily as the databases for research on the subject topic. Any sources published in a language other than English were excluded from the search results along with any sources whose results were not pertinent to the HF disease state.

For relevance and convenience, only full-text sources published within the last 10 years were included except the DIG-trial, since many of its sub analyses were conducted recent years. To rank the quality ratings of evidence, the JAMA Neurology website [16] was utilized and evidence was ranked from 1 to 5. Randomized controlled trials with proper power and systematic reviews with meta-analysis were ranked highest on the list, whereas opinions and case reports were ranked lowest. The literature search yielded 26 relevant articles that were analyzed for this review, including but not limited to randomized clinical trials, meta-analyses, and cohort studies. Of the 26 sources found through our literature search, the data represents an aggregate of 101,013 patients who were provided digoxin or placebo. Out of the 26 identified items, we found one multicenter randomized, controlled trial, 12 reviews, two clinical research studies, one multicenter prospective study, one observational study, three cohort studies, two prospective studies, two analyses, and one cluster analysis. 

## Review

The DIG trial [17] was a landmark study that investigated the effects of digoxin on rates of mortality and hospitalization rates among 6,800 stable HF patients with EF ≤ 40% with maintained normal sinus rhythms, regardless of having been given digoxin treatment prior to enrollment. In a double-blind fashion, patients were randomized in a 1:1 order for administration of a once-daily dose of digoxin or placebo, along with other guideline-based therapies. The patients in this trial were mainly Caucasian (nonwhite patients made up 14.4% of the digoxin group and 14.8% of the placebo group) and presented with New York Heart Association (NYHA) functional class II or III symptoms, with a mean age of 65 years. The trial population comprised primarily male patients, with only 22.2% female patients in the digoxin group and 22.5% in the placebo group. Almost 50% of the patients were on digoxin therapy during enrollment, and a high rate of utilization of angiotensin-converting enzyme (ACE)-inhibitor and diuretic therapies was observed among the participants. While no difference was observed for all-cause mortality across the digoxin and placebo groups, a lower risk for HF-based mortality was observed across both the groups in the form of a trend (p=0.06; Confidence Interval (CI)=95%, relative risk=0.88). Among the patients in the digoxin group, a notably lower rate of all-cause-, CV-, and HF-associated hospitalization was found. Despite the positive outcomes in hospitalization rates associated with digoxin use in the trial, the study had a few limitations, such as a limited diversity inpatient population in terms of sex and ethnicity, which could limit its generalizability. In nearly 50% of the placebo group patients randomized to digoxin, the drug was discontinued and > 20% of the patients may have been aware of the treatment they were receiving, thereby compromising the blinding in the study. Additionally, the mean serum drug concentration was observed to be 0.86 ng/mL and 0.80 ng/mL at the one-month and 12-month visits, respectively, which indicated a therapeutic serum drug concentration per current treatment standards [14,15], with no adverse effect of digoxin on survival [17].

The Heart Failure: A Controlled Trial Investigating Outcomes of Exercise Training (HF-ACTION) trial was conducted as a post-hoc analysis after the DIG trial. A dependence on multivariate adjustment was found for the association between digoxin use and a composite of all-cause mortality and hospitalization and secondary endpoints of composite of CV mortality or hospitalization due to HF [12].

Other post-hoc analyses of the DIG trial indicated from the database how the efficacy of digoxin falls into a narrow therapeutic window towards the lower end of the range and how a higher mortality rate may be attributed to its increase into the higher end of the normal range. The 0.25 mg daily dose of digoxin may be less beneficial autonomically and hemodynamically than that of the 0.125 mg dose. With increased serum digoxin concentration (SDC), there is a risk of higher intracellular calcium levels causing arrhythmias, which may affect any beneficial effects of digoxin in HF, a property that makes digoxin a challenging agent for therapy and calls upon the need to monitor its serum levels, along with those of potassium and other electrolytes. It is also imperative to monitor any drug-related interactions or adverse effects of digoxin that may or may not have an impact on serum creatinine and fluid levels [18,19].

It is also important to bear in mind that during the time when the DIG trial took place, agents like beta-blockers, mineralocorticoid receptor antagonists, as well as devices such as the cardioverter-defibrillator implants were not part of a guideline-based therapy and, hence, there may be some challenges as we try to understand the implications of the study in the current period [20]. 

Since very little was known about the effects of digoxin on mortality and hospital admission rates among patients with HFpEF, a clinical research study was organized to evaluate the 30-day and six-year outcomes of digoxin initiation before hospital discharge in patients of an older age. Out of the total 1026 subjects, 6% of the patients on digoxin and 9% patients who did not receive digoxin showed readmission within 30 days due to HF, but no association was found for 30-day or six-year outcomes among older HFpEF patients who received digoxin before being discharged from hospital stay [21].

A retrospective cohort study was carried out later. It included patients presenting with common comorbidities such as AF in their study to add more generalizability to the results obtained previously from the DIG trial. In this study, duration of hospitalization and overall mortality were found to be statistically insignificant across the groups at one, six, 12, and 24 months. Comparison of all-cause readmissions at six, 12, and 24 months suggested that digoxin treatment was attributed to an increase in respective rates of readmission at 53%, 34%, and 35%. While conducting analysis based on ethnicity, no benefit to the Caucasian patient population was observed, whereas use of digoxin in the Hispanic and African-American patient populations was associated with worsened or improved outcomes, respectively [22].

Another retrospective analysis of the DIG trial assessed the effects of digoxin in patients with mild systolic dysfunction of the left ventricle across three categories of patients with left ventricular EF: HFrEF (< 40%), HFmEF (40-49%), and HFpEF (≥ 50%). It was observed that digoxin played a key role in decreasing the composite of CV disease (CVD) or HF-related rehospitalization with a 95% CI (0.68-0.81), further corroborating with the findings of the DIG trial about digoxin having the most effect in HF hospitalization rates among patients with HFrEF [23].

A cluster analysis and a cluster-specific survival analysis of the DIG trial were also carried out to assess whether digoxin may be helpful or possess deleterious effects upon homogenous patient groups [24]. After classifying the DIG database into 20 clusters, the authors utilized Multivariate Cox regression analyses to evaluate whether an increase, decrease, or neutral effect was seen on all-cause mortality and HF readmissions from the use of digoxin. Digoxin was associated with an increase in mortality in patient groups composed of those who were of an older age, females, and those who had conditions such as hypertension, higher systolic blood pressure, increased heart rate, and increased EF. In terms of its effects on HF readmissions, per Kaplan-Meier analysis, digoxin was found to have had no associations (logrank $x^2$=0.5, p=0.5) among groups who had a higher number of female participants with hypertension (74% in the digoxin group and 69% in the placebo group), higher systolic blood pressure, increased BMI, higher EF, and decreased peripheral edema and third heart (S_3_) sound. Hence, the use of digoxin among female patient groups with hypertension, higher EF, and increased systolic blood pressure requires careful clinical consideration [24].

To assess the effects of digoxin discontinuation among ambulatory patients, a retrospective cohort study was initiated based on data obtained from the Organized Program to Initiate Lifesaving Treatment in Hospitalized Patients with Heart Failure (OPTIMIZE-HF) by linking it with data obtained from Medicare databases [25]. A cohort of 3,499 patients (mean age = 76 years) was identified to have been administered digoxin prior to admission, and the drug was discontinued among 721 patients prior to being discharged, and no associations were found between digoxin discontinuation and HF or all-cause readmission rates at 30 days after discharge. Digoxin was, however, found to be associated with increased risk related to all-cause mortality (p=0.001, CI=95% (1.26-2.57)). Another important finding of this study was the association among digoxin discontinuation, increased risks of HF, all-cause readmissions, and all-cause mortality at six and 12 months, suggesting poor outcomes occurring from the association between deleterious effects of digoxin to elderly patients hospitalized with HFrEF [25].

On the contrary, in an analysis of the effects of digoxin on mortality rates and CV hospitalizations from the Atrial Fibrillation and Congestive Heart Failure (AF-CHF) trial, digoxin was found to be attributed to a higher rate of all-cause mortality, arrhythmic deaths and CV mortality in patients presenting with HFrEF in combination with AF, whereas no impact was found on CV hospitalizations. The harmful effects of digoxin may not have any absolute causes but may be related to its pharmacokinetic properties that could result in toxic concentrations in a narrow range with the therapeutic levels [12]. A cohort study based on national registry data from China found that regardless of guideline-recommended therapy in HFrEF patients with or without AF, digoxin intake was associated with an increase in all-cause mortality and rehospitalization, but not HF mortality and rehospitalization. An explanation for this association was the increasingly morbid state of patients who are prescribed digoxin, with the presence of other possible non-CV disease states. Since the serum levels of digoxin were not monitored, limited data were available regarding possible digoxin toxicity [26].

## Conclusions

The use of digoxin in CVD states has come a long way since its discovery to its current place in guideline-based therapy for HFrEF as an agent for rate control in patients with AF. However, with its continued use, serious concerns persist regarding its potential toxicity and other deleterious effects, that may outweigh its benefits in the reduction of all-cause and CV hospitalization rates. Further studies are needed to define the appropriate role of digoxin in HF treatment.

## References

[1] Qamer SZ, Malik A, Bayoumi E (2019). Digoxin use and outcomes in patients with heart failure with reduced ejection fraction. Am J Med.

[2] Murphy SP, Ibrahim NE, Januzzi JL Jr (2020). Heart failure with reduced ejection fraction: a review. JAMA.

[3] Zhang L, Liebelt JJ, Madan N, Shan J, Taub CC (2017). Comparison of predictors of heart failure with preserved versus reduced ejection fraction in a multiracial cohort of preclinical left ventricular diastolic dysfunction. Am J Cardiol.

[4] Falletta C, Clemenza F, Klersy C (2019). Additive value of biomarkers and echocardiography to stratify the risk of death in heart failure patients with reduced ejection fraction. Cardiol Res Pract.

[5] Shah KS, Xu H, Matsouaka RA (2017). Heart failure with preserved, borderline, and reduced ejection fraction: 5-year outcomes. J Am Coll Cardiol.

[6] Clark KA, Velazquez EJ (2020). Heart failure with preserved ejection fraction: time for a reset. JAMA.

[7] Weiss AJ, Jiang HJ (2021). Overview of clinical conditions with frequent and costly hospital readmissions by payer, 2018: statistical brief #278. Healthcare Cost and Utilization Project (HCUP) Statistical Briefs [Internet].

[8] Tariq S, Aronow WS (2015). Use of inotropic agents in treatment of systolic heart failure. Int J Mol Sci.

[9] Ge Z, Li A, McNamara J, Dos Remedios C, Lal S (2019). Pathogenesis and pathophysiology of heart failure with reduced ejection fraction: translation to human studies. Heart Fail Rev.

[10] Maddox TM, Januzzi JL Jr, Allen LA (2021). 2021 Update to the 2017 ACC expert consensus decision pathway for optimization of heart failure treatment: answers to 10 pivotal issues about heart failure with reduced ejection fraction. J Am Coll Cardiol.

[11] Ambrosy AP, Bhatt AS, Stebbins AL (2018). Prevalent digoxin use and subsequent risk of death or hospitalization in ambulatory heart failure patients with a reduced ejection fraction-findings from the heart failure: a controlled trial investigating outcomes of exercise training (HF-ACTION) randomized controlled trial. Am Heart J.

[12] Elayi CS, Shohoudi A, Moodie E, Etaee F, Guglin M, Roy D, Khairy P (2020). Digoxin, mortality, and cardiac hospitalizations in patients with atrial fibrillation and heart failure with reduced ejection fraction and atrial fibrillation: an AF-CHF analysis. Int J Cardiol.

[13] Yancy CW, Jessup M, Bozkurt B (2017). 2017 ACC/AHA/HFSA focused update of the 2013 ACCF/AHA guideline for the management of heart failure: a report of the American College of Cardiology/American Heart Association task force on clinical practice guidelines and the Heart Failure Society of America. J Am Coll Cardiol.

[14] Ziff OJ, Lane DA, Samra M (2015). Safety and efficacy of digoxin: systematic review and meta-analysis of observational and controlled trial data. BMJ.

[15] McDonagh TA, Metra M, Adamo M (2021). 2021 ESC guidelines for the diagnosis and treatment of acute and chronic heart failure. Eur Heart J.

[16] (2021). JAMA Neurology. https://jamanetwork.com/journals/jamaneurology.

[17] Digitalis Investigation Group (1997). The effect of digoxin on mortality and morbidity in patients with heart failure. N Engl J Med.

[18] Chaggar PS, Shaw SM, Williams SG (2015). Is foxglove effective in heart failure?. Cardiovasc Ther.

[19] Konstantinou DM, Karvounis H, Giannakoulas G (2016). Digoxin in heart failure with a reduced ejection fraction: a risk factor or a risk marker?. Cardiology.

[20] Ambrosy AP, Butler J, Ahmed A, Vaduganathan M, van Veldhuisen DJ, Colucci WS, Gheorghiade M (2014). The use of digoxin in patients with worsening chronic heart failure: reconsidering an old drug to reduce hospital admissions. J Am Coll Cardiol.

[21] Lam PH, Packer M, Gill GS (2020). Digoxin initiation and outcomes in patients with heart failure with preserved ejection fraction. Am J Med.

[22] Alkhawam H, Abo-Salem E, Zaiem F (2019). Effect of digitalis level on readmission and mortality rate among heart failure reduced ejection fraction patients. Heart Lung.

[23] Abdul-Rahim AH, Shen L, Rush CJ, Jhund PS, Lees KR, McMurray JJ (2018). Effect of digoxin in patients with heart failure and mid-range (borderline) left ventricular ejection fraction. Eur J Heart Fail.

[24] Ather S, Peterson LE, Divakaran VG (2011). Digoxin treatment in heart failure-unveiling risk by cluster analysis of DIG data. Int J Cardiol.

[25] Malik A, Masson R, Singh S (2019). Digoxin discontinuation and outcomes in patients with heart failure with reduced ejection fraction. J Am Coll Cardiol.

[26] Zhou J, Cao J, Jin X (2020). Digoxin is associated with worse outcomes in patients with heart failure with reduced ejection fraction. ESC Heart Fail.

